# Size-dependent antioxidative activity of platinum nanoparticles

**DOI:** 10.1186/1753-6561-7-S6-P41

**Published:** 2013-12-04

**Authors:** Hidekazu Nakanishi, Takeki Hamasaki, Tomoya Kinjo, Kiichiro Teruya, Shigeru Kabayama, Sanetaka Shirahata

**Affiliations:** 1 Division of Life Engineering, Graduate School of Systems Life Sciences, Kyushu University, Fukuoka 812-0053, Japan; 2 Department of Bioscience and Biotechnology, Faculty of Agriculture, Kyushu University, Fukuoka 812-0053, Japan; 3 Nihon Trim Co. Ltd, Osaka 531-0076, Japan

## Background

So far, most of studies on nanometer-sized metal particles have focused on biological safety and potential hazards. However, anti-oxidative activity of noble metal nanoparticles (NPs) attracts much attention, recently. Platinum nanoparticles (Pt NPs) are one of the most important noble metals in nanotechnology because Pt NPs have negative surface potential from negative charges and are stably suspended from an electric repulsion between the same charged particles [1]. We previously reported that Pt NPs of 2-3 nm sizes scavenged reactive oxygen species (ROS) such as superoxide anion radical, hydrogen peroxide and hydroxyl radical *in vitro *[2]. Here, we report the cytotoxicity and size-dependent antioxidative activity of Pt NPs on rat skeletal muscle cell line, L6.

## Materials and methods

Pt NPs were synthesized by a modified citrate reduction method of Hydrogen hexachloroplatinate (IV). Particle size and concentrations of Pt NPs were determined by a transmittance electron microscope (TEM) and ICP-MS, respectively. To find the toxic effect of Pt NPs rat myoblast L6 cells were pre-cultured for 24 hours in culture medium with a 10^-3 ^to 10 mg/l of Pt NPs and cell viability was determined by WST-1 assay. To investigate the anti-oxidative effect of Pt NPs on L6 cells, the relative amount of intracellular H_2_O_2 _was measured with a Bes-H_2_O_2_-AC florescent probe, which is designed to detect intracellular H_2_O_2 _specifically [3]. The intracellular ROS levels when treated with 1 mg/l of Pt NPs for 2 hours were measured using IN Cell Analyzer 1000.

## Results and conclusions

**Figure 1:**
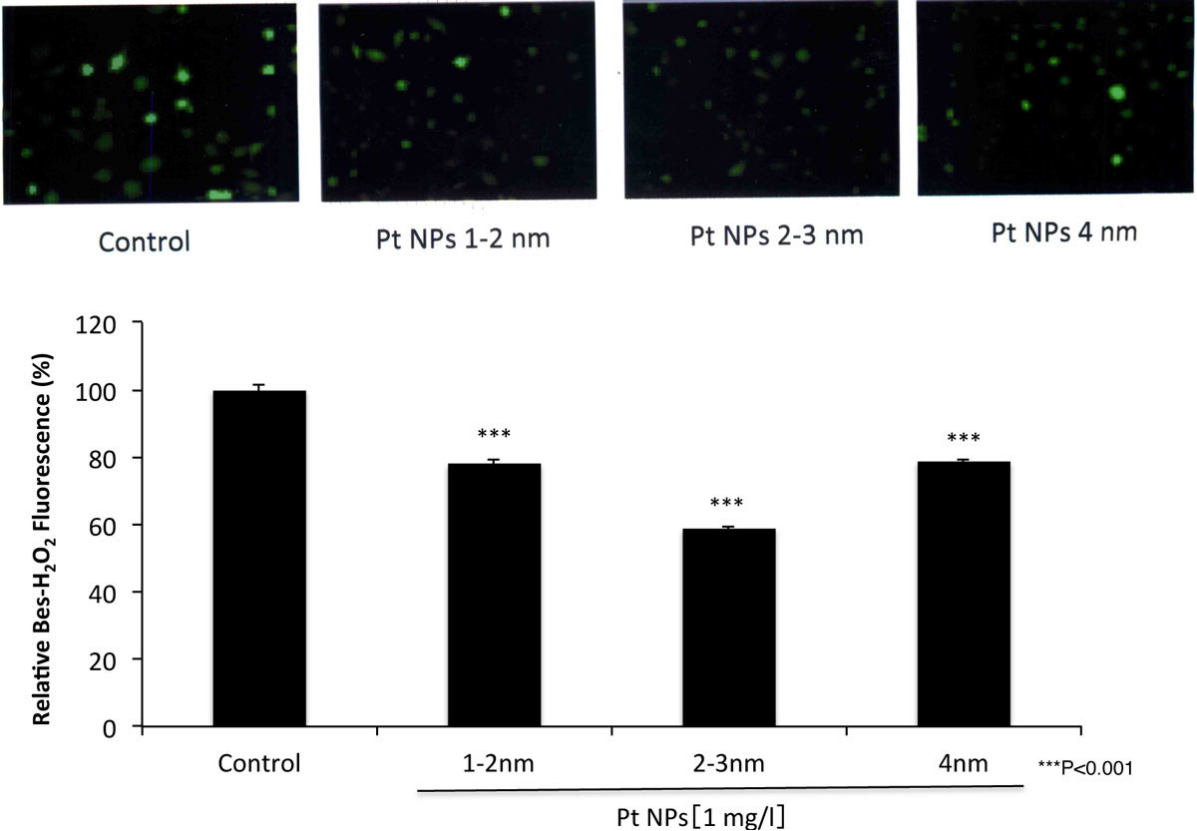
**The scavenging effect of several sized Pt NPs on intracellular hydrogen peroxide in L6 cells**. Asterisks donate significant difference from the untreated control cells. (***P < 0.001).

The particle sizes we synthesized were determined to 1-2 nm, 2-3 nm and 4 nm respectively (data not shown). Cytotoxicity of Pt NPs of these sizes was not observed at a concentration below 10 mg/l (data not shown). Intracellular ROS levels are thought to result from a primary response to internalized NPs leading to decreased cell viability [4]. Thus, the suppression of excess ROS is of prime importance for cell survival. The intracellular ROS levels were decreased significantly by the whole sizes of Pt NPs treatment and 2-3 nm of Pt NPs scavenged the ROS most efficiently (Figure 1). The relative fluorescence level treated with 2-3 nm of Pt NPs decreased significantly to about 60% (*** P < 0.001) compared with that of non-treated cells. Smaller NPs should be more taken up by the cells efficiently and might more scavenge ROS effectively [5]. However, the Pt NPs of 1-2 nm less scavenged the intracellular ROS than that of 2-3 nm. The one reason might be that 1-2 nm of Pt NPs is rather too small to activate intracellular anti-oxidant defense pathways than 2-3 nm of Pt NPs because of their less cytotoxicity. However, we have no data to show. Therefore, we have to make more effort to investigate the relationship between the sizes of Pt NPs and ROS scavenging activity.

Our results suggest Pt NPs of 2-3 nm sizes have no cytotoxity below 10 mg/l and are useful materials to scavenge ROS. In this regard Pt NPs are expected as redox regulation factors for suppression of various ROS-related diseases.
